# Average direct costs of outpatient, hospital, and home care provided to patients with chronic wounds^*^

**DOI:** 10.1590/1980-220X-REEUSP-2022-0295en

**Published:** 2022-11-14

**Authors:** Paula Buck de Oliveira Ruiz, Antônio Fernandes Costa Lima

**Affiliations:** 1 Universidade de São Paulo, Escola de Enfermagem, Departamento de Orientação Profissional, São Paulo, SP, Brazil.

**Keywords:** Wounds and Injuries, Nursing, Costs and Cost Analysis, Cost Control, Health Care Costs, Direct Service Costs, Heridas y Lesiones, Enfermería, Costos y Análisis de Costo, Control de Costos, Costos de la Atención en Salud, Costos Directos de Servicios, Ferimentos e Lesões, Enfermagem, Custos e Análise de Custo, Controle de Custos, Custos de Cuidados de Saúde, Custos Diretos de Serviços

## Abstract

**Objective:**

To analyze the average direct costs of outpatient, hospital, and home care provided to patients with chronic wounds.

**Method:**

Quantitative, exploratory-descriptive case study, carried out in a Comprehensive Wound Care Unit. Costs were obtained by multiplying the time spent by professionals by the unit cost of labor in the respective category, adding to the costs of materials and topical therapies.

**Results:**

Outpatient care costs corresponded to US$4.25 (SD ± 7.60), hospital care to US$3.87 (SD ± 17.27), and home care to US$3.47 (SD ± 5.73). In these three modalities, direct costs with dressings and medical consultations were the most representative: US$7.76 (SD ± 9.46) and US$6.61 (SD ± 6.54); US$7.06 (SD ± 24.16) and US$15.60 (SD ± 0.00); US$4.09 (SD ± 5.28) and US$15.60 (SD ± 0.00), respectively.

**Conclusion:**

Considering comprehensive care for patients with chronic wounds, the mean total direct cost was US$10.28 (SD ± 17.21), with the outpatient modality being the most representative in its composition. There was a statistically significant difference (p value = 0.000) between the costs of home and outpatient, home and hospital, and outpatient and hospital care.

## RESUMO

**Objetivo:**

Analisar os custos diretos médios da assistência ambulatorial, hospitalar e domiciliar prestada aos pacientes com feridas crônicas.

**Método:**

Pesquisa quantitativa, exploratório-descritiva, do tipo estudo de caso, realizada numa Unidade de Tratamento Integral de Ferida. Obtiveram-se os custos multiplicando-se o tempo despendido pelos profissionais pelo custo unitário da mão de obra da respectiva categoria, somando-se aos custos dos materiais e terapias tópicas.

**Resultados:**

Os custos da assistência ambulatorial corresponderam a US$4,25 (DP ± 7,60), da hospitalar a US$3,87 (DP ± 17,27) e da domiciliar a US$3,47 (DP ± 5,73). Nessas três modalidades, os custos diretos com os curativos e consultas médicas foram os mais representativos: US$7,76 (DP ± 9,46) e US$6,61 (DP ± 6,54); US$7,06 (DP ± 24,16) e US$15,60 (DP ± 0,00); US$4,09 (DP ± 5,28) e US$15,60 (DP ± 0,00), respectivamente.

**Conclusão:**

Considerando a assistência integral aos pacientes com feridas crônicas, o custo direto médio total foi de US$10,28 (DP ± 17,21), sendo a modalidade ambulatorial a mais representativa na sua composição. Constatou-se diferença estatística significante (valor p = 0,000) entre os custos da assistência domiciliar e ambulatorial, domiciliar e hospitalar e ambulatorial e hospitalar.

## RESUMEN

**Objetivo:**

Analizar los costos directos promedios de la atención ambulatoria, hospitalaria y domiciliaria prestada a pacientes con heridas crónicas.

**Método:**

Investigación cuantitativa, exploratoria-descriptiva, del tipo estudio de caso, realizada en una Unidad de Atención Integral de Heridas. Los costos se obtuvieron al multiplicar el tiempo dedicado por los profesionales por el costo unitario de la mano de obra en la respectiva categoría, sumado a los costos de materiales y terapias tópicas.

**Resultados:**

Los costos de atención ambulatoria correspondieron a US$ 4,25 (DE ± 7,60), hospitalaria a US$ 3,87 (DE ± 17,27) y atención domiciliaria a US$ 3,47 (DE ± 5,73). En estas tres modalidades, los costos directos con apósitos y consultas médicas fueron los más representativos: US$7,76 (DE ± 9,46) y US$6,61 (DE ± 6,54); US$7,06 (DE ± 24,16) y US$15,60 (DE ± 0,00); US$4,09 (DE ± 5,28) y US$15,60 (DE ± 0,00), respectivamente.

**Conclusión:**

Considerando la atención integral a pacientes con heridas crónicas, el costo directo promedio total fue de US$ 10,28 (DE ± 17,21), siendo la modalidad ambulatoria la más representativa en su composición. Hubo una diferencia estadísticamente significativa (p-valor = 0,000) entre los costos de atención domiciliaria y ambulatoria; domiciliaria y hospitalaria y, por fin, ambulatoria y hospitalaria.

## INTRODUCTION

The world population’s aging and the increase in the incidence of chronic diseases highlight the need to improve care provided to people with wounds. However, for the implementation of effective care, greater awareness is required of the growing clinical challenge associated with wounds, as well as the development of an appropriate methodology to ensure that clinical practices are employed with the best cost-benefit ratio^(1)^.

Among the types of wounds, chronic wounds are those that do not progress through a normal, orderly, and timely repair sequence, resulting from disordered healing mechanisms^(2)^, including pressure injuries (PI), diabetic foot ulcers (DFU), venous ulcers and arterial ulcers^(3)^. Based on causal etiologies, the *Wound Healing Society* classifies chronic wounds into four categories: PI, diabetic ulcers, venous ulcers, and arterial insufficiency ulcers^(4)^.

In developed countries, chronic wounds affect 1% to 2% of the population. It is estimated that up to 4.5 million people carry them in the United States of America (USA)^(5,6)^. A meta-analysis study showed a combined prevalence of 2.21/1000 individuals for chronic wounds of mixed etiologies and an estimated prevalence of 1.51/1000 individuals for chronic leg ulcers^(7)^. It should be noted that due to the increase in life expectancy, the non-communicable chronic diseases, the resistance of microorganisms, and the occurrence of biofilm, the wounds associated with these factors will also increase and, consequently, will increase the costs related to their treatment, significantly impacting economic-financial aspects of health institutions^(8)^.

It is estimated that the costs resulting from the care of patients with chronic wounds can vary between 1% and 3% of expenses related to health^(6)^. However, this value may be underestimated, given that current studies report higher costs, as there are variables that have not yet been considered, such as decreased productivity and quality of life of patients and early retirements^(9)^.

Research carried out in the USA, using the health insurance system *Medicare,* demonstrated that wounds affected 15% of users and estimated the annual cost to $28 billion^(10)^. In Brazil, a study conducted in a palliative and long-term care unit identified the costs of materials required for PI dressings, estimating that the average total cost was BRL 36,629.95/month and BRL915.75 patient/month, projecting that costs could reach BRL 445,664.38/year^(11)^.

The treatment of people with chronic wounds is an economic burden for the health system, imposes an important financial burden on society, and impairs patients’ quality of life and of work^(12)^. In this perspective, nurses can develop research identifying the costs associated with the treatment of patients with chronic wounds, in different care contexts, contributing to the effective allocation and efficient use of the required resources.

Research on human and economic resources to allow the treatment of chronic wounds is scarce. Thus, it is essential to produce knowledge about the financial repercussions of chronic wounds for the individual and the healthcare system^(1)^. Thus, to support the decision-making process of administrators, managers, and health professionals, the present study was carried out to analyze the average direct costs (ADC) of outpatient, hospital, and home care provided to patients with chronic wounds.

## METHOD

### Design of Study

Quantitative, exploratory-descriptive, single-case study type research.

### Population

Treatments/care provided to patients with PI, diabetic ulcer (DU), and vasculogenic ulcer (VU) in a Comprehensive Wound Care Unit (CWCU), in 2019 (N = 5,241).

### Local

The CWCU provides care in three modalities: outpatient (at its own headquarters), intra-hospital (in three partner hospitals: A, B and C), and home. Located in the Southwest region of Bahia, BA, Brazil, it provides interdisciplinary, outpatient, and hospital care, from Monday to Friday from 8:00 am to 6:00 pm, hospital nursing care; on weekends, from 7:00 am to 12:00 pm, through a shift schedule among nurses; and home care, from Monday to Friday, from 7 am to 7 pm, and on weekends, from 7 am to 1 pm. The materials, drugs, and solutions required are planned and provided exclusively by the CWCU.

The staff consists of five nurses, a nursing technician (NT), four physicians (an intensivist specialized in hyperbaric oxygen therapy, a psychiatrist, an orthopedist, and a dermatologist), a nutritionist, an administrative professional, and a receptionist. The nursing team is divided into a coordinator, responsible for administrative services and reference in home care; one nurse in charge of quality and auditing; three assistant nurses, one for care at the headquarters and at hospital B and the other two directed to care at hospitals A and C; and a NT that provides home care. The medical team has medical partners in vascular and cardiology specialties, who provide care through referrals and scheduling at the CWCU’s headquarters.

In 2019, the CWCU performed 5,241 treatments/services to patients with PI, DU and VU, financed by five Health Plan Operators, private patients, and the Brazilian Public Health System (*SUS*).

### Selection Criteria

Patients with PI, DU, and VU, over 18 years of age, assisted in the CWCU, who have started outpatient, hospital, and/or home treatments/care were selected to integrate the study. Patients with acute injuries were excluded.

### Sample Definition

Faced with the Covid-19 pandemic and the challenge of ensuring the feasibility of this study, a statistician established the sample size of the number of observations required to calculate the outpatient, hospital, and home care ADCs, based on the number of treatments/services performed by the CWCU in 2019 (N = 5,241). He calculated a minimum sample through the sum of the population of the places where the patients were treated, considering 95% confidence and 5% error.

Then, non-participant observations of 65 dressings, 65 documentation from the Systematization of Nursing Care (*SAE*), and 10 medical consultations in outpatient care; 64 dressings, 64 *SAE* documentation on hospital care; and 68 dressings and 68 *SAE* documentation in home care were carried out. The number of medical consultations observations in home care was not established, since in the CWCU, when the patient needed this type of assistance, he/she was referred to the outpatient clinic (headquarters). However, to face the challenges of the Covid-19 pandemic, the CWCU started carrying out telemedicine medical consultations also to patients in home care. Therefore, we chose to observe this variation in the form of care provided to home patients. It should be clarified that, in the CWCU, nurses perform the nursing consultation concomitantly with the dressing and, for this reason, it was not possible to record both of them separately.

### Data Collection

**Chart 1:**
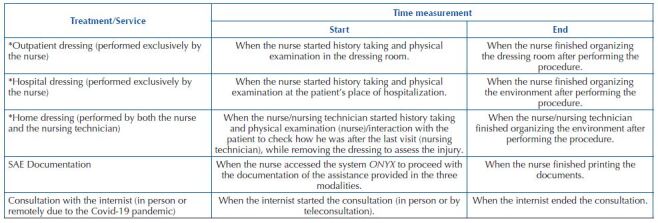
Establishment of the beginning and end of the measurement of the time spent by the health professional during the care of patients with chronic wounds in the CWCU – Vitória da Conquista, BA, Brazil, 2020.

Data collection took place in March, August, and September/2020, with the professional category, the number of professionals involved, and time spent (timed) in outpatient, hospital and home care, as well as the use of materials, drugs, solutions, and specific products for dressings (topical therapies) being documented. The beginning and end of the measurement of the time spent to perform treatments/attendance were standardized as specified in Chart 1.

*Nursing consultation occurs concomitantly with the dressing; for this reason, dismembering them during non-participant observations was not possible.

The values for ADC calculation of health professionals’ direct labor (DL), of materials and specific products for dressings were obtained from the nurse auditor of the CWCU Billing and Accounting Service.

To measure ADCs related to health professionals, the average salaries (salaries, benefits, bonuses and social charges) were used, according to the respective professional category, multiplying the time spent (timed) by the DL unit cost^(13)^. In the CWCU, the number and volume of materials/solutions are standardized according to the size (ratio between size × width) of the dressing and are classified as: small – *kit S* (5.0 × 5.0cm), medium – *kit* M (above 5.1 × 5.1cm up to 10.0 × 10.0cm) and large – *kit L* (above 10.1 × 10.1cm). Values related to materials and topical therapies corresponded to the average price paid for the replacement of the last three acquisitions.

Total ADC was calculated by multiplying the (measured) time spent by health professionals in the execution of treatments/attendance by the DL unit cost, adding to the cost of materials and solutions/drugs^(14)^. The values were presented in US dollars (US$), and the conversion carried out at the rate of US$1.00/BRL5.28 (rate on 09/18/2020, provided by the Brazilian Central Bank).

### Data Analysis and Treatment

To calculate ADCs, the analyses were obtained through the *SPSS Statistics* (Version 23), with the descriptive and inferential functions being performed to check probability issues of a population based on the sample data. The sample profile was described, contemplating the analyzed variables and their consequences, replicating the data in an absolute and relative way.

In the inferential scope, the independence and prediction between the variables proposed in the scope of the study were analyzed using, within the expected standards, the Spearman Correlation, Mann-Whitney U test, Kruskal-Wallis test, and Wilcoxon test. The results were obtained through the analysis of p-value, considering <0.05 as significance between the groups studied. The tests contemplate an alpha error of 5% and a reliability of 95%.

### Ethical Aspects

After consent of the CWUC’s Board of directors, the study was approved by the Research Ethics Committee of the proposing institution (opinion number 3.781.012 of 12/18/2019). The legal procedures established by Resolution No. 466, of December 12, 2012, which approves the guidelines and regulatory standards for research involving human beings, were strictly followed.

## RESULTS

We observed 60 patients assisted by the CWCU, 30 males and 30 females, aged between 24 and 93 years, mean of 72.1 years (SD ± 13.4); 24 (40.0%) patients received home care; 19 (31.7%) hospital care, and 17 (28.3%) outpatient care.

A total of 85 (100.0%) chronic wounds were recorded, ranging from one to four per patient, with a mean of 1.42 (SD ± 0.7); distributed in 34 (40.0%) PI, 34 (40.0%) VU, and 17 (20.0%) DU; 20 (33.3%) patients had two or more chronic wounds. Twenty-four patients, with 35 wounds, were treated at home; 19, with 26 wounds, in hospitals A, B and C; and 17, with 24 wounds, at the CWCU’s main outpatient clinic.

Among the 228 (100.0%) treatments/attendance observed, 84 (36.8%) occurred at home, 77 (33.8%) in the clinic, and 67 (29.4%) in hospitals; 228 (100%) SAE documentations, 84 (36.8%) at home, 77 (33.8%) at the outpatient clinic, and 67 (29.4%) at hospitals; and 43 (100.0%) medical consultations (in person and telemedicine). Due to the pandemic, some in-person medical appointments that would be held in the clinic or hospitals were cancelled, others reduced or carried out remotely. Thus, 19 (44.2%) telemedicine consultations were carried out for patients receiving home care; 21 (48.8%) consultations for outpatients (14–66.7% in person and 7–33.3% by telemedicine); and three (7.0%) telemedicine consultations for patients in hospital care.

During the study period, 55 (64.7%) chronic wounds required small dressings, with PI being the most representative (29 dressings – 52.7%), followed by VU (19 dressings – 34.6%), and DU (7 dressings – 12.7%). For 20 (23.5%) chronic wounds, medium dressings were performed, with the amount of dressings for VU (12-60.0%), DU (5–25.0%), and PI (3–15, 0%) being highlighted. Among the 10 (11.7%) large dressings, five (50.0%) were performed for the treatment of DU, three (30%) for VU, and two (20%) for DU. When using the Kruskal-Wallis test, there was a statistically significant association between the size of the dressings and non-classifiable PI (p = 0.034) and DU (p = 0.044). Significance was found between the type of care and PI stage 2 (p = 0.012), PI stage 3 (p = 0.025) and VU (p = 0.036).

Five nurses, a nursing technician (NT) and an internist performing outpatient care (at the CWCU), hospital care (hospitals A, B and C), and home care were observed. The average age of these professionals corresponded to 29 years (SD ± 7.0), the average time of training to nine years (SD ± 5.4), and the average time of work in the CWCU to four years (SD ± 5.4). Among nursing professionals, age ranged from 24 to 40 years, with a mean of 28 (SD ± 6.3) years and with training time from one to 13 years, with a mean of 5.5 (SD ± 5.1)) years. Some nurses had specialization courses in ICU nursing, emergency and urgency (two), dermatotherapy (one), and specific courses such as laser therapy (one), podiatry (one), and hyperbaric oxygen therapy (one). The internist had residency in ICU, specialization in hyperbaric oxygen therapy.

ADC calculation of the comprehensive care provided to patients with PI, DU, and VU covered the performance of dressings (variables: cost with personnel [Nurse and TE’s DL], cost with material and cost with topical therapy), SAE documentation (variable: cost with personnel [Nurse’s DL]), and medical consultations (variable: cost with personnel [physician’s DL]).

The costs of materials/solutions that are part of the *kits* totaled US$1.12, using micropore, and US$1.18, using bandage, for the *kit* S; US$1.43, using micropore and US$1.58 using bandage, for the *Kit M*; and US$1.96 using micropore, and US$2.16 using bandage, for the *kit* L.

In comprehensive outpatient care, the mean total time was 8.93 minutes (SD ± 5.20), ranging from 1.00 to 30.00 minutes. For the dressing, the average time corresponded to 11.10 minutes (SD ± 5.90), with a minimum of 4.00 and a maximum of 31 minutes; the medical consultation took 12.71 minutes (SD ± 12.58), ranging from 2.00 to 30.00 minutes; and SAE documentation 2.99 minutes (SD ± 1.96), ranging from 1.00 to 12 minutes.

As shown in Table 1, total ADC for comprehensive outpatient care was US$4.25 (SD ± 7.60), ranging from US$0.03 to US$28.59. The most representative value was the dressing (US$7.76 – SD ± 9.46) and the most expressive variable, the ADC with topical therapy (US$5.98 – SD ± 9.15), followed by materials/solutions (*kits*) (US$1.45 – SD ± 0.41). Total ADC of the medical consultation was US$6.61 (SD ± 6.54), the second procedure that most contributed to the composition of total ADC of outpatient care.

**Table 1.** Distribution of observations of outpatient procedures performed on patients with chronic injuries, in March, August and September 2020, according to the ADC with personnel, materials/solutions (*kits*), topical therapy, and ADC total care – Vitória da Conquista, BA, Brazil, 2020.

Variable	Mean	SD±	Median	min–max
Nurse’s DL – US$	0.33	0.18	0.30	0.12–0.0.93
ADC with personnel – US$	0.33	0.18	0.30	0.12–0.0.93
ADC with materials/solutions (*kits*) – US$	1.45	0.41	1.18	1.12–2.16
ADC with Topical Therapy – US$	5.98	9.15	1.48	0.17–25.51
**Total ADC of the dressing (n = 77) – US$**	7.76	9.46	2.99	1.47–28.59
**Total ADC – SAE Documentation (Nurse’s DL) (n = 77) – US$**	0.09	0.06	0.09	0.03–0.36
**Total ADC – Medical Consultation (Physician’s DL) (n = 21) – US$**	6.61	6.54	2.08	1.04–15.6
**Total ADC of comprehensive outpatient care – US$**	4.25	7.60	1.59	0.03–28.59

*Exchange rate: BRL5.28/US$1.00, based on the rate on 09/18/2020, according to the Central Bank.

In comprehensive hospital care, the average total duration corresponded to 13.40 minutes (SD ± 14.63), ranging from 2.00 to 30.00 minutes. Mean time for the dressing was 9.87 minutes (SD ± 5.02), ranging from 3.00 to 33.00 minutes; for the medical consultation, it was 30.00 minutes (SD ± 0.00), and for the SAE documentation it was 5.16 minutes (SD ± 2.49), ranging from 1.00 to 9.00 minutes.

As shown in Table 2, total ADC for comprehensive hospital care was US$3.87 (SD ± 17.27), ranging from US$0.06 to US$199.58. The most relevant variables were the ADC of the medical consultation, US$15.60 (SD ± 0.00) and of the dressing with US$7.06 (SD ± 24.16), highlighting the impact of ADC with topical therapy (US$5.35 – SD ± 24.07) and materials/solutions (*kits*) (US$1.40 – SD ± 0.33).

**Table 2.** Distribution of observations of hospital procedures performed on patients with chronic injuries, in March, August and September 2020, according to the ADC with personnel, materials/solutions (*kits*), topical therapy, and ADC total care – Vitória da Conquista, BA, Brazil, 2020.

Variable	Mean	SD±	Median	min–max
Nurse’s DL – US$	0.31	0.18	0.30	0.09–0.99
ADC with personnel – US$	0.31	0.18	0.30	0.09–0.99
ADC with materials/solutions (*kits*) – US$	1.40	0.33	1.18	1.12–2.16
ADC with Topical Therapy – US$	5.35	24.07	1.23	0.04–197.41
**Total ADC of the dressing (n = 67) – US$**	7.06	24.16	2.89	1.29–199.58
**Total ADC – SAE Documentation (Nurse’s DL) (n = 67) – US$**	0.15	0.07	0.12	0.06–0.54
**Total ADC – Medical Consultation (Physician’s DL) (n = 3) – US$**	15.60	0.00	15.60	15.60–15.60
**Total CDM of comprehensive hospital care – US$**	3.87	17.27	1.45	0.06–199.58

*Exchange rate: BRL5.28/US$1.00, based on the rate on 09/18/2020, according to the Central Bank.

In comprehensive hospital care, the average total duration corresponded to 13.40 minutes (SD ± 14.63), ranging from 34.00 to 67.00 minutes. Mean time for the dressing was 7.80 minutes (SD ± 4.07), ranging from 3.00 to 28.00 minutes; for the medical consultation it was 30.00 minutes (SD ± 0.00), and for the SAE documentation it was 2.39 minutes (SD ± 2.49), ranging from 1.00 to 9.00 minutes.

As shown in Table 3, total ADC for comprehensive hospital care was US$3.47 (SD ± 17.27), ranging from US$0.03 to US$28.38. The most significant ADCs were those of medical consultation (US$15.60 – SD ± 0.00) and dressing (US$4.09 – SD ± 5.28), a procedure whose composition of the ADC was determined notably by the costs with the topical therapy (US$2.61 – SD ± 5.11) and with materials/solutions (*kits*) (US$1.24 – SD ± 0.22).

**Table 3.** Distribution of observations of outpatient procedures performed on patients with chronic injuries, in March, August and September 2020, according to the ADC with personnel, materials/solutions (*kits*), topical therapy, and ADC total care – Vitória da Conquista, BA, Brazil, 2020.

Variable	Mean	SD±	Median	min–max
Nurse’s DL – US$	0.27	0.12	0.21	0.12–0.84
Nursing technician’s DL – US$	0.22	0.13	0.21	0.09–0.48
ADC with personnel – US$	0.23	0.12	0.21	0.09–0.84
ADC with materials/solutions (*kits*) – US$	1.24	0.22	1.12	1.12–2.16
ADC with Topical Therapy – US$	2.61	5.11	0.49	0.03–25.51
**Total ADC of the dressing (n = 84) – US$**	4.09	5.28	1.95	1.39–28.39
**Total ADC – SAE Documentation (Nurse’s DL) (n = 82) – US$**	0.11	0.18	0.06	0.03–1.36
**Total ADC – Medical Consultation (Physician’s DL) (n = 19) – US$**	15.60	0.00	15.60	15.60–15.60
**Total ADC of comprehensive home care – US$**	3.47	5.73	1.59	0.03–28.38

*Exchange rate: BRL5.28/US$1.00, based on the rate on 09/18/2020, according to the Central Bank.

Considering the comprehensive care provided by the CWCU, the total ADC was US$10.28 (SD ± 17.21). Table 4 shows that ADC with outpatient care was US$4.25 (SD ± 7.60), US$3.87 (SD ± 17.27) for hospital care, and US$3.47 for home care (SD ± 17.27). Among the analyzed groups, ADCs in the home and outpatient modalities present a statistically significant difference between them (p value = 0.000). When analyzing the total ADC between the home and hospital modalities, a statistical difference was found, by the significant method (p value = 0.000); the same occurred when comparing the groups analyzed between total ADC of outpatient and hospital modalities (p = 0.000).

**Table 4.** Distribution of observations of treatments/care provided to patients with chronic injuries, in March, August and September 2020, in outpatient, hospital and home modalities, according to the respective total ADC and total comprehensive care ADC – Vitória da Conquista, BA, Brazil, 2020.

Variable	Mean	SD±	Median	min–max	p value
**Total ADC of the dressing (n = 77) – US$**	7.76	9.46	2.99	1.47–28.59	pdxa = 0.306 pdxh = 0.424 paxh = 0.328
**Total ADC of the dressing (n = 67) – US$**	7.06	24.16	2.89	1.29–199.58	
**Total ADC of home dressing (n = 84) – US$**	4.09	5.28	1.95	1.39–28.39	
**Total ADC Outpatient SAE Documentation (n = 77) – US$**	0.09	0.06	0.09	0.03–0.36	pdxa = 0.454 pdxh = 0.000 paxd = 0.000
**Total ADC Hospital SAE Documentation (n = 67) – US$**	0.15	0.07	0.12	0.06–0.54	
**Total ADC Household SAE Documentation (n = 82) – US$**	0.11	0.18	0.06	0.03–1.36	
**Total ADC – Outpatient Medical Consultation (n = 21) – US$**	6.61	6.54	2.08	1.04–15.6	pdxa = 0.001 pdxh = 1.000 paxh = 0.083
**Total ADC – Hospital Medical Consultation (n = 3) – US$**	15.60	0.00	15.60	15.60–15.60	
**Total ADC – Home medical consultation (n = 19) – US$**	15.60	0.00	15.60	15.60–15.60	
**Total ADC of comprehensive outpatient care – US$**	4.25	7.60	1.59	0.03–28.59	pdxa = 0.000 pdxh = 0.000 paxh = 0.000
**Total ADC of hospital care – US$**	3.87	17.27	1.45	0.06–199.58	
**Total ADC of comprehensive home care – US$**	3.47	17.27	1.45	0.06–199.58	

*Exchange rate: BRL5.28/US$1.00, based on the rate on 09/18/2020, according to the Central Bank.

## DISCUSSION

Chronic wounds are a burden to the health financial system and, with the increase in the elderly population, as an example the USA, their occurrence could reach 77 million people in 2060^(13)^. Associated with Chronic Noncommunicable Diseases, chronic wounds will result in significant increases in quantity and associated costs^(14)^.

The measurement of the ADC of comprehensive care provided by the CWCU to patients with chronic wounds, in the outpatient, hospital and home modalities corresponded to US$10.28, US$4.25 referring to outpatient care, US$3.87 to the hospital and US$3.47 at home. The relevance of studies aiming to analyze this kind of costs, which are still scarce, is highlighted, and from them to subsidize the rational allocation of inputs with a view to more efficient and effective results. Research related to the estimation of costs with wound care worldwide indicates that, in 2022, the values may extrapolate US$ 15 billion, driven by the increase in the incidence of chronic wounds, technological advances, and the increase in population’s life expectancy^(15)^.

When analyzing each care modality, the highest total ADCs were observed in the outpatient clinic, with ADCs of dressings (US$7.76 – SD ± 9.46) being highlighted and topical therapy (US$5.98 – SD ± 9.15) being the most representative variable. A study of the financial transfer from the *SUS* regarding the procedures performed on an outpatient basis indicated that 6,101 were related to dressings, corresponding to US$207,941.80, and the dressing classified as grade II (unna’s boot) showing the most impactful total ADC from the use of materials and/or solutions^(16)^.

Therapeutic changes have favored out-of-hospital care. With the anticipation of hospital discharges, outpatient care is growing, putting greater pressure on this care modality in terms of the volume of care provided and respective costs. In the 2000s, in the USA, treatments/care for people with chronic and complex wounds were mostly in the hospital. However, over the years, outpatient care centers have been developed, but this is still an innovation in development, requiring knowledge of the costs involved, so that adequate investment and reimbursement goals can be established, providing subsidies for integral and effective^(10)^ care.

In hospital care, the most relevant total ADCs were those of medical consultation (US$15.60 – SD ± 0.00) and dressing (US$7.06 – SD ± 24.16), with ADCs with topical therapy (US$5.35 – SD ± 24.07) being highlighted. It should be reiterated that, with the decree regarding the closing of borders due to the Covid-19 pandemic, CWCUs needed to invest in adjustments to allow the continuity of care, through telemedicine. In an Israeli study, which compared the cost of distance care (telemedicine) and in-person care, it was pointed out that the former was 7.0% higher, but when proportionally analyzing telemedicine and in-person care, the cost of telemedicine was smaller^(17)^. Thus, the most significant ADC presented in hospital care related to medical consultations may be related to the beginning of telemedicine care in the CWCU, and adaptations regarding this form of care are still necessary.

Regarding the high costs of topical therapy, a research conducted in an inpatient unit of a university hospital, when measuring the total ADC of dressings (ADC and other supplies), showed that the most significant values corresponded to materials and solutions^(18)^. It is worth mentioning the indispensability of health professionals knowing the different therapeutic options and their respective costs, regardless of the type of chronic wound, because with the limitation of resources designated for health and the growing increase in expenses and costs, adopting conducts based on evidence can help them in the effectiveness of the treatment. The importance and repercussion of the consumption of material resources in hospital costs is evidenced, which can also be compromised by ineffective management, reaching between 23% and 30% of the budget of public or private health organizations^(19)^.

In home care, ADC for medical consultation (US$15.60 – SD ± 0.00) and dressing (US$4.09 – SD ± 5.28) were the highest. Due to the context of the Covid-19 pandemic, home visits had to be replaced by consultations via telemedicine, increasing the costs related to their adequacy in the work process at the CWCU, impacting the costs of this care modality. Telemedicine aims to coordinate and integrate care and requires the use of tools that meet the needs of health professionals and patients, so that it can, in fact, favor comprehensive care. Therefore, the costs involved have to be analyzed and detailed^(20)^, to support the decision-making process regarding its implementation.

A survey with patients treated at a nursing home in Australia obtained the total ADC of AU$98,489.22, with the costs related to PI prevention equipment being explained, followed by dressings, medical consultations and additional medications. It showed lower costs when compared to those obtained in the present study, but the Australian researchers remark that the variables “topical therapy” and “professional workforce” would need to be reviewed and adequate, using multidisciplinary practices and being duly based on evidence^(21)^. In this regard, the provision of quality care to patients requires financial investments for organizational adequacy to ensure the allocative efficiency of the resources applied to health care^(22)^.

ADCs analysis of the comprehensive care for patients with chronic wounds, provided by a CWCU based on standardized protocols, will provide health professionals, administrators and managers with in-depth knowledge of the economic and financial aspects associated with outpatient, hospital, and home care modalities. Such knowledge will help in decision-making, care, and management, and may support future studies that propose to use the same methodology for calculating costs.

## CONCLUSION

The total ADC of comprehensive care provided by the CWCU to patients with PI, DU, and VU corresponded to US$10.28 (SD ± 17.21), with US$4.25 (SD ± 7.60) in the outpatient modality, US$3.87 (SD ± 17.27) at hospital, and US$3.47 (SD ± 17.27) at home. There was a significant statistical difference between the costs in the home and outpatient (p value = 0.000); home and hospital (p value = 0.000); and outpatient and hospital (p value = 0.000) modalities.
